# Risk factors for development of chronic periodontitis in Bulgarian patients (pilot research)

**DOI:** 10.1080/13102818.2014.974328

**Published:** 2014-11-12

**Authors:** Maria Stoykova, Nina Musurlieva, Doychin Boyadzhiev

**Affiliations:** ^a^ Deparment of Social Medicine and Public Health, Faculty of Public Health, Medical University of Plovdiv, Plovdiv, Bulgaria; ^b^ Department of Applied Mathematics and Modeling, Faculty of Mathematics and Informatics, Plovdiv University ‘Paisii Hilendarski’, Plovdiv, Bulgaria

**Keywords:** diabetes, odds ratio, periodontitis, risk factors, stress

## Abstract

The aim of this work was to assess the risks and analyse the risk factors for development of chronic periodontitis in Bulgarian patients. The quality of life was investigated in a cohort of 228 patients with chronic periodontitis. Within the frame of this study, pilot research (a case-control study) was conducted among 80 patients (20 cases and 60 control patients without periodontitis) to evaluate the risk for development of chronic periodontitis. The minimum sample size of patients was determined based on power analysis for sample-size calculation. The mean age of participants in the control group was 31.33 ± 9.38 years and in the case group, 33.00 ± 11.52. Data were accumulated by clinical and sociological methods. Descriptive statistics and multi-factor logistic regression analysis (Backward Conditional procedure) were used. One-factor dispersion analysis showed that, of the 12 studied risk factors, the following variables were significant: stress, diabetes, presence of calculus, overlapping and misaligned teeth (*P* < 0.05). Multiple logistic regressions were applied to evaluate the association between the variables. Three predictors were selected in the final logistic regression equation: diabetes (*B* = 4.195; *P* = 0.001), crooked and overlapping teeth (*B* = 3.022; *P* = 0.010) and stress (*B* = 2.882; *P* = 0.014). The logistic risk assessment model for development of periodontitis has a predictive value of 93.80% (χ^2^ = 63.91; *P* = 0.000). Our results confirmed some proven risk factors for periodontal disease. In the studied population, diabetes was the single, most important predictor for development of periodontitis.

## Introduction

Periodontitis is a disease of multi-factor etiology. The major etiological factor seems to be the accumulation of bacterial plaque. However, not all bacterial plaque accumulations result in periodontal disease.[1] Therefore, other risk factors also exist. They seem to modulate the immune response of the organism to the periodontal infection and determine the level of its susceptibility to it.[2] Controversy surrounds the classification of risk factors for periodontitis. Clerehugh et al. [3] distinguish between three main groups of risk factors for development of periodontitis: (1) local factors, e.g. poor tooth restoration, overlapping teeth, mouth breathing, muscle parafunctions, etc.; (2) systemic factors, e.g. smoking, diabetes, genetic disorders, stress, systemic diseases, AIDS, medication use, poor diet, age, sex, educational and socio-economic status, etc. and (3) risk indicators (potential risk factors), e.g. obesity, osteoporosis, low calcium levels, osteopenia. Their presence is associated with increased risk of periodontitis.[3] Spanish authors differentiate age, sex and socio-economic status as determinants of risk.[4]

In Bulgaria, data on the epidemiology of periodontal infection are scarce and there is no periodontitis register. The limited available data on periodontitis risk factors in Bulgaria, as well as the controversy in different authors’ opinion, prompted this study.

This work was aimed at assessment of risk and analysis of risk factors for development of periodontitis in the studied patient population.

## Subjects and methods

### Subjects

From November 2010 to February 2011, a study was conducted to assess the quality of life in 228 patients with chronic periodontitis. Participants were randomly selected from outpatients at the Department of Periodontology, Faculty of Dentistry, Medical University of Plovdiv (Bulgaria) and from various dentist surgeries in Plovdiv. All of them had sought treatment in the department and the dentist surgeries.

Within the frame of the above-mentioned research, a pilot case-control study was conducted, including 80 patients (20 cases and 60 controls). It aimed to assess the risk of chronic periodontitis in the selected patients. The minimum sample size of patients was determined based on power analysis for sample-size calculation. Age under 20 years was an exclusion criterion. The mean age of the participants was 31.33 ± 9.38 for the control group and 33.00 ± 11.52 for the case group.

### Clinical method used for selecting the cases and controls

The periodontal health status was measured using The Community Periodontal Index (CPI) recommended by the World Health Organization (WHO) as a standard epidemiological examination method for periodontal disease, with dental mirror and WHO periodontal probe. Periodontitis was diagnosed based on the presence of clinically significant inflammation, peridontal osseous pockets and bone destruction. Thus, we selected 60 controls (randomly selected patients without periodontitis who had visited the dentist surgeries for other reasons) and 20 cases out of 228 patients included in the primary survey. The cases included patients with chronic periodontitis who were matched to the controls, according to the most important socio-demographic parameters: sex, age and education. The aim was to eliminate the impact of obscuring factors. The case/control ratio was 1:3.

### Sociological method

A direct, individual interview was performed with the participants from the cases and the control group. From the basic interview questionnaire (developed to assess the quality of life of the 228 patients with chronic periodontitis), only the sections related to risk factors assessment were used. The relevant sections were: socio-demographic characteristics of the patients cohort and presence of common risk factors prior to the development of periodontitis: smoking, alcohol abuse, stress, diabetes, vegetarian diet, fresh vegetable and fruit consumption, medication use, presence of hazards in the professional environment, level of oral hygiene, tooth clenching and grinding, regular calculus removal procedures, presence of overlapping and crooked teeth and regularity of dental examinations.

### Statistical assessment

Descriptive statistics was used: analysis of variance, alternative analysis and non-parametric analysis (Fisher's exact test, *t*-test, Pearson criterion). Multiple logistic regressions were applied to evaluate the association between the variables (backward conditional procedure). Statistical significance was assumed at *P* ≤ 0.05. SPSS version13.0 was applied for data processing.

In the process of data analysis, the categorical variables were recoded into dichotomous variables. The dependent variable was given two values: healthy subjects were assigned a value of ‘1’ and patients were assigned a value of ‘0’. Independent variables were coded in a similar manner. For instance, the independent variable smoking was assigned two values: negative response was coded as ‘1’, meaning absence of periodontitis, and all positive responses were coded as ‘0’. Only the variable regular fruit and vegetable consumption was coded in the reverse manner: regular consumption (positive response) was coded as ‘1’ and negative response was coded as ‘0’.

## Results and discussion

No statistically significant difference was observed in the socio-demographic parameters between cases and controls. The percentages according to sex, age and education are presented in Table 1.

**Table 1.** Socio-demographic characteristics of cases and controls.

	Controls		Cases			
Characteristics	*n*	%	*n*	%	Criterion	*p*
Education						
Secondary	34	56.67	10	50.00	χ^2^ = 2.400	0.493
High	26	43.33	10	50.00		
Total	60	100.00	20	100.00		
Age						
Mean age ± SD	31.33 ± 9.38		33.00 ± 11.52		*t* = 0.420	0.518
Sex						
Male	26	43.33	9	45.00	χ^2^ = 0.017	0.896
Female	34	56.67	11	55.00		
Total	60	100.00	20	100.00		

Most people in the studied sample were young adults: the mean age of controls was (31.33 ± 9.38) years, the mean age of cases was (33.00 ± 11.52) years. The number of females was slightly higher than that of males: the former were 56.67% of the patients in the control group and 55% in the cases group. The control group was comprised mostly of patients with secondary education (56.67%). In the cases group, the percentage of patients with higher education was equal to that of patients with secondary education.

In order to determine the strength of predictor variables for development of chronic periodontitis in the studied contingent, the effect size of each variable was investigated separately. Non-parametric analysis (Fisher's test) was used. The odds ratio (*OR*) and the 95% confidence interval (CI) for each of the 12 variables was calculated.

The statistical analysis (Table 2) showed that the following variables were statistically non-significant (*P* > 0.05) and they were excluded: smoking (*P* = 0.349), alcohol use (*P* = 0.151), vegetarian diet (*P* = 0.162), diet (lack of fresh fruit and vegetables in the daily menu) (*P* = 0.485), occupational hazards (*P* = 0.080), oral hygiene (*P* = 0.225), tooth clenching and grinding (*P* = 0.081). The significant variables (*P* < 0.05) included: diabetes, stress, crooked and overlapping teeth, dentist practices visits and calculus removal.

**Table 2.** Strength of predictor variables in the studied sample.

	Groups						
	Controls		Cases				
Variable	*n*	%	*n*	%	*P*	*OR*	95% CI
Smoking	28	46.67	9	45.00	0.35	1.39	0.50/3.86
Alcohol use	34	56.67	8	40.00	0.15	0.51	0.18/1.43
Hazards	3	5.00	1	5.00	0.08	0.19	0.02/1.56
Stress	45	75.00	12	60.00	0.00	33.00	8.43/129.19
Vegetarian diet	13	21.67	1	5.00	0.16	3.35	0.62/18.16
Diet (lack of fresh fruit and vegetables in the daily menu)	10	16.67	4	20.00	0.48	0.80	0.22/ 2.90
Oral hygiene (teeth brushing less than once daily)	7	11.67	5	25.00	0.22	0.29	0.03/ 2.51
Calculus removal (none)	9	15.00	14	70.00	0.00	13.22	4.02/ 43.47
Teeth clenching and grinding	10	16.67	7	35.00	0.08	2.69	0.85/ 8.43
Crooked and overlapping teeth	12	20.00	15	75.00	0.00	12.00	3.63/ 39.58
Diabetes	4	6.67	17	85.00	0.00	79.33	16.14/ 389.92
Dentist practices visits (no visits or less than one visit annually)	14	23.33	18	90.00	0.00	0.03	0.01/0.16

Multiple logistic regressions (backward conditional procedure) were used to assess the combined impact of the selected significant variables (via one-factor analysis).

Following the multiple regression construct, three significant predictor variables were selected in the final equation: diabetes, stress, crooked and overlapping teeth. These factors showed the strongest relative weight in the studied cohort. The variable calculus removal is not present in the final equation (*P* = 0.800).

Based on the accumulated empirical data, a logistic model was constructed to assess the risk of periodontitis development. It incorporates the three selected variables: diabetes, stress, crooked and overlapping teeth. The model was shown to have a 93.80% predictive value (χ^2^ = 63.91; *P* = 0.001). Diabetes had the strongest predictive value (highest regression coefficient).(1) 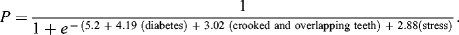



The selected predictor variables for development of periodontitis (Table 2) have been studied in a number of other research works.[2,5–9] None of the 80 interviewed patients in the case-control study indicated the use of therapeutic agents such as Verapamil, Nifedipin, Diltiazem, hydantoin medication and Cyclosporin А. For this reason, medication use (which has been studied as a factor by other authors) could not be investigated in our study. In the available reports, there is data that the above-mentioned therapeutic agents are related to the development of periodontitis.[10] In our study, only diabetes – as a systemic disorder – was incorporated in the statistical modelling, as none of the interviewed patients had indicated suffering from other genetic or immune-deficient disorders.

Interestingly, in the studied population, smoking was not selected as a risk factor for development of periodontitis. On the other hand, a number of other researchers have indicated cigarette use as one of the most important predictors for periodontitis.[11,12] Unlike their results, in the cohort studied by us, no statistically significant difference was established between the percentages of smokers in the control and the cases group: 46.66% ± 6.44% smokers in the cases group and 45.00% ± 11.12% in the control group. Our results indicated that in the studied population, smoking was not a significant factor for development of periodontitis (*P* = 0.908). This is likely due to the fact that our cohort was comprised mostly of young adults: 82.50% ± 4.24% of the patients belonged to the 20–39 age group. It seems that in these patients the duration of tobacco use was not long enough to induce a harmful effect on the periodontal tissues.

Similar results were obtained when the effect of alcohol use was studied. There was a statistically non-significant difference (*P* = 0.247) between the cases (56.67% ± 6.40%) and the controls (35.00% ± 10.66%). Therefore, alcohol did not prove to be a significant risk factor in our cohort. In similar cases, most authors recommend a more detailed investigation of the role of alcohol use in the pathogenesis of periodontitis. They focus on the correlation between frequency and severity of periodontitis and the frequency of alcohol consumption, the duration of alcohol consumption and the type of alcohol use (hard drinks versus beer and wine).[13] Nonetheless, our study documents the negative trend typical of the Bulgarian society at present for a wider spread of two harmful habits, smoking and alcohol use.

The microorganisms in the tooth plaque are considered the major etiological factor inducing the pathological changes in the periodontal tissues.[1,14,15] The magnitude of plaque accumulations are largely dependent on the level of oral hygiene. Most patients in the studied group maintained good oral hygiene, only one person in each group indicated that they had never brushed his/her teeth (*P* = 0.69): in the control group (1.67% ± 1.65%) and in the cases group (5.00% ± 4.87%). Thus, poor level of oral hygiene was not proven a risk factor for periodontitis in the studied cohort.

Most Bulgarians share a traditional diet rich in meat and meat products. The current western trends for vegetarian diet are not yet widely spread in the Bulgarian society. Only five patients declared that they are vegetarians. Therefore, we could not establish a correlation between periodontitis development and vegetarian diet (*P* = 0.162). Similarly, no correlation was established between periodontitis and low consumption of fresh vegetables and fruit (*P* = 0.485).

In people exposed to occupational hazards (presence of chemical, physical and biological agents in the work environment), there is a higher frequency and severity of periodontal diseases.[15] A study with participants exposed to chemical and physical hazards in the city of Plovdiv (Bulgaria), reported the following incidence of periodontal diseases: 100% in zinc plant workers versus 99.01% in lead production workers.[15] In our study, the percentage of patients with a history of exposure to professional hazard was very low: 5.00% ± 2.81% in the control group and 5.00% ± 4.87% in the cases group. Therefore, in the studied cohort, exposure to professional hazards was not a significant predictor of periodontal disease (*P* = 0.080) most probably due to the small number of patients with such history that fell in the selected sample.

Based on Fisher's exact test, dentist practices visits were selected as a significant predictor of periodontitis. The calculated *OR* < 1 indicates that dental surgeon visits are not a risk factor, but have a protective effect against periodontitis in the studied cohort. Therefore, dentist practices examinations could aid the prophylaxis of periodontal diseases.

Periodontitis is a disease of multi-factor etiology and requires investigation of the complex effect of the risk factors. Logistic regression analysis can be applied to analyse the effect size and to determine the independent predictors for the development of periodontitis. The results from the regression analysis showed that three independent predictors of periodontitis can be selected: diabetes, stress and crooked and overlapping teeth (Table 3). Diabetes as a risk factor had the highest regression coefficient (*B*); thus, it had the strongest effect on the development of periodontitis. Similarly, other authors also document that periodontal disease is more frequent and severe in diabetic patients.[16,17] The second highest regression coefficient, after diabetes, was that of crooked and overlapping teeth. Stress was ranked third. Calculus removal, as a risk factor, was not present in the final equation (*P* = 0.800). Based on these findings, and the important role of diabetes as a risk factor, we recommend a closer cooperation between dental practitioners, endocrinologists and general practitioners, aimed at prevention, early detection and diagnosis of periodontal diseases.

**Table 3.** Multi-factor regression model of the predictor variables in the studied sample.

Variable	*B* (regression coefficient)	SD (standard deviation)	*p*
Diabetes	4.195	1.222	0.001
Crooked and overlapping teeth	3.022	1.118	0.010
Stress	2.882	1.236	0.014
Const.	−5.270	1.302	0.000

Based on the three predictor variables with the strongest effect in the studied sample, a model of risk assessment was constructed. This model determined the least number of predictors (risk factors) that have the strongest predictor value in the development of periodontitis. The model was shown to have 93.80% predictive significance (χ^2^ = 63.91; *P* = 0.001). This construct allows the dentists to undertake an adequate prophylactic approach in patients who have a history of risk factors for peridontitis, e.g. diabetes, crooked and overlapping teeth and stress.

A limitation of this pilot study is the small number of participants. Further investigation of the risk factors would need a more representative sample of patients. Future research should also include patients from various age groups, as mostly young adults were included in this study. This would allow estimating the effect of factors such as smoking and alcohol use.

Taken together, our results support the general understanding that integrated preventive strategies based on the common risk factors approach are recommended for public health practice.[18] The national health authorities should ensure, therefore, that prevention of periodontal diseases is made an integral part of the prevention of diabetes and other chronic diseases, as well as of health promotion.[18,19]

## Conclusions

Our study included patients who reported exposure to some of the common risk factors for the development of periodontitis: diabetes, stress, crooked and overlapping teeth, poor oral hygiene, tobacco use, excessive alcohol consumption. The obtained results provided confirmatory evidence for the presence of risk factors for peridontitis. In our contingent, diabetes had a strongest predictive value in the development of the disease. Based on these findings, we recommend a closer cooperation between dental practitioners, endocrinologists and general practitioners, aimed at prevention, early detection and diagnosis of periodontal diseases. In order to investigate the impact of all factors for periodontitis described in the available literature, a larger number of patients of various age groups are needed.
